# Mediating effect of resilience between social support and compassion fatigue among intern nursing and midwifery students during COVID-19: a cross-sectional study

**DOI:** 10.1186/s12912-023-01185-0

**Published:** 2023-02-15

**Authors:** Jia-Ning Li, Xiu-Min Jiang, Qing-Xiang Zheng, Fen Lin, Xiao-Qian Chen, Yu-Qing Pan, Yu Zhu, Ru-Lin Liu, Ling Huang

**Affiliations:** 1 School of Nursing, Fujian Medical University, Fuzhou City, Fujian Province China; 2 Fujian Maternity and Child Health Hospital College of Clinical Medicine for Obstetrics & Gynecology and Pediatrics, Fujian Medical University, Fuzhou City, Fujian Province China; 3 Fujian Obstetrics and Gynecology Hospital, Fuzhou City, Fujian Province China; 4 Fujian Medical University Union Hospital, Fuzhou City, Fujian Province China; 5 School of Nursing, Fujian University of Traditional Chinese Medicine, Fuzhou City, Fujian Province China

**Keywords:** Compassion fatigue, COVID-19 pandemic, Intern nursing and midwifery students, Resilience, Social support

## Abstract

**Aims:**

To examine the mediating effect of resilience between social support and compassion fatigue among intern nursing and midwifery students during COVID-19.

**Background:**

Compassion fatigue has become exceedingly common among intern nursing and midwifery students, especially during the COVID-19 pandemic. Social support and resilience can help intern nursing and midwifery students control their negative emotions, reduce compassion fatigue, and increase their well-being. However, the mediating effect of resilience between social support and compassion fatigue remains unclear.

**Design:**

A multicentre cross-sectional survey.

**Methods:**

A total of 307 intern nursing and midwifery students were recruited from November 2020 to February 2021 in tertiary grade A hospitals in China. Structural equation modelling was applied to analyse the mediating effects of resilience between social support and compassion fatigue. The Social Support Rating Scale, the 10-item Connor-Davidson Resilience Scale, and the Chinese version of the Compassion Fatigue Short Scale were used to collect data. The hypothetical path model was tested by using IBM SPSS version 26.0 and AMOS version 26.0 software.

**Results:**

Intern nursing and midwifery students had moderate compassion fatigue. Social support positively affected resilience (*β* = 0.514, *p* < 0.01). Social support negatively affected compassion fatigue (*β* = − 0.310, *p* < 0.01), while resilience negatively affected compassion fatigue (*β *= − 0.283, *p* < 0.01). Resilience played a mediating role between social support and compassion fatigue.

**Conclusion:**

Social support can directly affect the compassion fatigue of intern nursing and midwifery students during COVID-19 and indirectly through resilience. Stronger resilience can reduce compassion fatigue. Accordingly, resilience-based interventions should be developed to reduce compassion fatigue.

**Supplementary Information:**

The online version contains supplementary material available at 10.1186/s12912-023-01185-0

## Introduction

Compassion fatigue is a type of burnout experienced by caregivers supporting suffering individuals [1]. Figley defined compassion fatigue as “the cost of caring” [2]. Compassion fatigue was associated with a wide range of physical and emotional symptoms, including irritability, anxiety, depression, frustration, insomnia, and pain [3, 4]. Most health care providers, such as palliative care providers, hospice health care practitioners, physicians, midwives, nurses, and nursing students, are at risk of developing compassion fatigue [5]. Although compassion fatigue has been studied extensively in the nursing profession, few studies have been conducted among nursing or midwifery students [6, 7]. Care providers appear to be especially sensitive and susceptible during this period of transition from student to professional [8]. During their internship, students are frequently exposed to stressful workplaces and traumatic situations similar to professional nurses [6], which may shape compassion fatigue at the start of their clinical placement. Therefore, their compassion fatigue should also be a concern, as it is vital to ensure the formation of an emotionally healthy nursing workforce.

The COVID-19 pandemic is the most serious global public health emergency in a century, which potentially contributes to compassion fatigue [9, 10]. In this context, intern nursing and midwifery students experienced additional stress, such as infection anxiety, greater workload, and shortage of protective equipment [11]. In addition, emotional ups and downs were heightened by witnessing patients infected, quarantined and dying in front of them [12]. Their compassion fatigue may not be apparent; however, it may later affect the quality of nursing and even the development of professional identity [13, 14]. Furthermore, Stamm suggested that the presence of vulnerability factors (such as a lack of social support) and personal strengths (such as resilience) may explain the development of compassion fatigue [15]. Therefore, a better understanding of the specific mechanisms underlying the development of compassion fatigue during internships can allow for targeted prevention in the future.

Social support represents the emotional, psychological, or physical support provided by individuals or organizations, which has certain effects on an individual’s mental health [16]. Previous studies found that nurses suffering from compassion fatigue felt depressed and isolated [17]. Support from social networks helped them recover from compassion fatigue [18]. The lockdown strategy initiated in China controlled the rapid spread of COVID-19. Meanwhile, physical distancing also prevented young students from interacting with their peers, resulting in social isolation and emotional exhaustion [19, 20]. Furthermore, during transition from campus to hospital, they have difficulty adjusting to complex and dynamic social relationships [21]. Therefore, intern nursing and midwifery students need sufficient social support to help them continue their clinical placement during COVID-19 [22].

Resilience refers to an individual’s capability to bounce back from traumatic events. During the COVID-19 pandemic, resilience allowed nurses to rebound successfully [23]. Previous studies revealed the value of resilience in countering various psychological effects in nurses [24, 25]. Furthermore, as an important mediator, resilience significantly decreased the negative effects of the ongoing pandemic on nurses’ mental health [9, 26, 27]. Resilience has been considered a protective resource against compassion fatigue, and its key factor was social support [28]. However, to our knowledge, no studies have examined the mediating effects of resilience between social support and compassion fatigue among intern nursing and midwifery students, despite growing evidence highlighting the role of resilience during the pandemic. Hence, in this study, we investigated the mediating role of resilience between social support and compassion fatigue among nursing and midwifery students during the COVID-19 pandemic. The following hypotheses were proposed:

Hypothesis 1 Social support positively correlates with resilience.

Hypothesis 2 Resilience negatively correlates with compassion fatigue.

Hypothesis 3 Social support negatively correlates with compassion fatigue.

Hypothesis 4 Resilience mediates the relationship between social support and compassion fatigue.

### Theoretical framework

This study is guided by the Compassion Fatigue Model [29], which has implications for nursing and midwifery practice. According to this model, a lack of resources, an absence of positive feedback, and reactions to personal distress expose nurses to the danger of compassion fatigue [29]. The COVID-19 pandemic poses a significant challenge to the resources of intern nursing and midwifery students, and insufficient resources (e.g., lack of resilience, inadequate social support) result in compassion fatigue. In this study, we examined the mediating effect of resilience between social support and compassion fatigue among intern nursing and midwifery students during COVID-19.

## Methods

### Design

This study was a multihospital study utilizing a cross-sectional survey and adhered to the Strengthening the Reporting of Observational Studies in Epidemiology (STROBE) guidelines (see Appendix S1) [30]. The sample size for this study was calculated based on a popular and public calculator for structural equation models [31]. The minimum sample size for detecting the effect is 147, and for the model structure, it is 200. (Anticipated effect size: 0.3; Desired statistical power level: 0.95; Number of latent variables: 2; Number of observed variables: 6; Probability level: 0.05). Hence, the minimum sample size is 200.

All participants were recruited from two universities in Fujian Province, China, and interned in seven tertiary grade A hospitals from November 1st, 2020, to February 28th, 2021. There were 85,424 confirmed cases of COVID-19 with 4634 deaths when the survey started and 89,912 confirmed cases with 4636 deaths when the survey ended. During this study, the COVID-19 pandemic had already lasted for a year since the initial outbreak at the end of 2019, in the city of Wuhan, China. Although China contained the spread of the outbreak, new cases continued to emerge occasionally [32]. Fourth-year intern nursing and midwifery students were allowed to participate in final-year clinical internship practice.

The inclusion criteria were as follows: (a) studying in a nursing and midwifery program at BSc. level; (b) having undergone clinical placement at least 3 months during COVID-19 pandemic; and (c) understanding the content of this study and volunteering to participate in this study. The exclusion criteria were as follows: (a) having various psychiatric disorders, including melancholia, severe anxiety, mania, and bipolar affective disorder, diagnosed by clinicians according to the diagnostic guidelines for psychiatric disorders; and (b) having been on sick leave for more than 1 month.

### The questionnaires

An online questionnaire was used to collect data in this study. All questionnaires were displayed in the Chinese language.

#### Socialdemographic questionnaire

The sociodemographic questionnaire was prepared by the researchers based on a literature review [33, 34], including gender, whether the participant was the only child, family’s location, whether the participant’s relatives working in the nursing profession, subject in high school, and the per monthly income of the family members.

#### Chinese version of the compassion fatigue short scale(C-CFSS)

The Chinese version of the Compassion Fatigue Short Scale, translated by Sun et al., has good psychometric properties for use in studies with Chinese medical workers [35]. It consists of two subscales: (a) a 5-item secondary trauma scale, and (b) an 8-item burnout scale. A total of 13 items were scored on a 10-item Likert scale ranging from 1 (never) to 10 (very frequently). We categorized the degree of compassion fatigue into three levels: low (scores between 13 and 51), medium (scores between 52 and 91), and high (scores between 92 and 130) [36]. The Chinese version shows an appropriate reliability with a Cronbach’s α of 0.90 [37]. Cronbach’s α coefficient for this study was 0.954.

#### Social support rating scale (SSRS)

The Social Support Rating Scale is widely adopted in China [38]. It is commonly used to assess social support in Chinese populations and its reliability and validity have been confirmed [38]. The 10-item scale evaluates 3 dimensions: objective support, subjective support, and usage of support. The total score of social support is the sum of scores from the 10 items. The objective support score is the sum of Items 2, 6 and 7; the subjective support score is the sum of Items 1 and 3 to 5, and the usage of support is the sum of Items 8 to 10. Items 1 to 5 and 8 to 10 were rated on a 4-point Likert scale, ranging from 1 (not at all) to 4 (very much). For Items 6 and 7, if the response is “No source” is answered, a score of 0 is given; if “have a source”, each source provides 1 point. Overall, the higher the score, the greater the level of individual social support. The Cronbach’s α of the Chinese scale is 0.89–0.94 [38]. Cronbach’s α coefficient for this study was 0.710.

#### 10-item Connor-Davidson resilience scale (CD-RISC-10)

The 10-item Connor-Davidson Resilience Scale was used to measure resilience in university nursing students. It captures core features of resilience over the preceding month. Each item is rated on a five-point Likert scale ranging from 0 “never” to 4 “almost always”, with higher scores indicating greater resilience [39]. The scale has been tested in undergraduate samples and student nurses with satisfactory reliability and validity [39, 40]. The Chinese version is developed based on nursing students and demonstrates good reliability with a Cronbach’s α of 0.851 [41]. Cronbach’s α coefficient for this study was 0.936.

### Data collection

The study was previously approved by two universities. The questionnaire survey was collected using Questionnaire Star (a tool for questionnaire surveys) via WeChat. Participants were recruited through a convenience sampling method and included intern nursing and midwifery students from the two universities. After obtaining informed consent from participants, the investigators explained the method of filling out questionnaires to participants by using questionnaire guidance language. Participants are guaranteed anonymity and confidentiality. They can withdraw freely from the study at any time and for any reason.

### Data analysis

IBM SPSS 26.0 and AMOS 26.0 software were used to analyse the data. The enumeration data were described as percentages (%), and the measurement data were described using the mean ± standard deviation. All data were checked for normality using QQ-plots and histograms and were found to be approximately normally distributed. Pearson correlation analysis was used to analyse the relationships among social support, resilience and compassion fatigue scores. All tests were two-sided, and *p* value less than 0.05 were regarded as statistically significant.

To evaluate the fitness of the hypothetical model, the chi-square/degrees of freedom ratio (χ^2^/*df*), root mean square error of approximation (RMSEA), goodness-of-fit index (GFI), adjusted goodness-of-fit index (AGFI), normed fit index (NFI), incremental fit index (IFI), Tucker-Lewis index (TLI) and comparative fit index (CFI) were used. The following threshold values were recommended as criteria for an adequate model: χ^2^/*df* < 3.00, RMSEA < 0.08, GFI > 0.80, AGFI > 0.80, NFI > 0.90, IFI > 0.90, TLI > 0.90 and CFI > 0.90 [42].

## Results

### Participants’ sociodemographic characteristics

A total of 307 valid responses were returned out of the 325 questionnaires that were distributed (representing a 94.5% response rate). Ninety-two percent of nursing and midwifery students were female and 7.8% were male. Among them, 233 cases (75.9%) had siblings, families of 182 cases (59.3%) reside in the urban area, and 243 cases (79.2%) had no relatives working in the nursing profession. The other sociodemographic characteristics of intern nursing and midwifery students are shown in Table 1.

**Table 1:** Sociodemographic characteristics of participants (*N* = 307)

Variables	***N***(%)
**Gender**	
Male	24(7.8%)
Female	283(92.2%)
**The only child**	
Yes	74(24.1%)
No	233(75.9%)
**family’s location**	
Rural	125(40.7%)
Urban or Suburban	182(59.3%)
**Subject in high school**	
Major in liberal arts	96(31.3%)
Major in science	211(68.7%)
**Relatives working in the nursing profession**	
Yes	64(20.8%)
No	243(79.2%)
**Family per monthly income (Yuan, RMB)**	
<5000	54(17.6%)
5000–8000	140(45.6%)
>8000	113(36.8%)

### The mean score of social support, resilience and compassion fatigue

The participants on average scored 31.61 ± 5.88 on the Social Support Rating Scale. The scores for objective support, subject support and usage of support were 6.97 ± 2.63, 16.94 ± 3.25, and 7.70 ± 1.81, respectively. The mean score of the Connor-Davidson Resilience Scale was 24.49 ± 6.64, while the mean score of the Chinese version of the Compassion Fatigue Short Scale was 60.61 ± 24.93, the “Secondary trauma” dimension score was 21.97 ± 10.80, and the “Burnout” dimension score was 38.64 ± 15.33. All results are shown in Table 2.

**Table 2:** The comparison of SSRS, CD-RISC-10, and C-CFSS Score (*N* = 307)

Variables	Mean ± SD
SSRS (potential point:12–66)	31.61 ± 5.88
Objective support (potential point:1–22)	6.97 ± 2.63
Subjective support (potential point:8–32)	16.94 ± 3.25
Usage of support (potential point:3–12)	7.70 ± 1.81
CD-RISC-10(potential point:0–40)	24.49 ± 6.64
C-CFSS (potential point:13–130)	60.61 ± 24.93
Secondary trauma (potential point:5–50)	21.97 ± 10.80
Burnout (potential point:8–80)	38.64 ± 15.33

Abbreviation: (1) *SSRS* the scores of the Social Support Rating Scale (2) *CD-RISC-10* the score of the 10-item Connor-Davidson Resilience Scale (3) *C-CFSS* the score of the Chinese version of the Compassion Fatigue Short Scale

### Correlation analysis of social support, resilience and compassion fatigue

Table 3 reveals that resilience was negatively correlated with compassion fatigue and its dimensions (*p* < 0.01). Resilience was positively correlated with social support and subjective support (*p* < 0.01). However, it was weakly correlated with objective support and usage of support (*p* < 0.01). Compassion fatigue was negatively correlated with social support and subjective support (*p* < 0.01). No significant relation was found between compassion fatigue and objective support (*p* = 0.134).

**Table 3:** Pearson correlation coefficients among variables (*N* = 307)

Variables	SSRS	OS	SS	US	CD-RISC-10	C-CFSS	ST	BO
SSRS	1							
OS	.744^**^	1						
SS	.843^**^	.376^**^	1					
US	.655^**^	.285^**^	.397^**^	1				
CD-RISC-10	.417^**^	.250^**^	.386^**^	.298^**^	1			
C-CFSS	−.312^**^	−0.086	−.358^**^	−.248^**^	−.454^**^	1		
ST	−.210^**^	−0.026	−.258^**^	−.183^**^	−.356^**^	.934^**^	1	
BO	−.360^**^	−.121^*^	−.401^**^	−.275^**^	−.487^**^	.968^**^	.815^**^	1

Abbreviation: (1) *SSRS* the scores of Social Support Rating Scale, *OS* the scores of objective support, *SS* the scores of subjective support, *US* the scores of usage of support (2) *CD-RISC-10* the score of the 10-item Connor-Davidson Resilience Scale (3) *C-CFSS* the score of the Chinese version of the Compassion Fatigue Short Scale, *ST* the score of secondary trauma, *BO* the score of burnout

*p<.05 **p<.01

### Fitness of the hypothetical path model

**Fig. 1:**
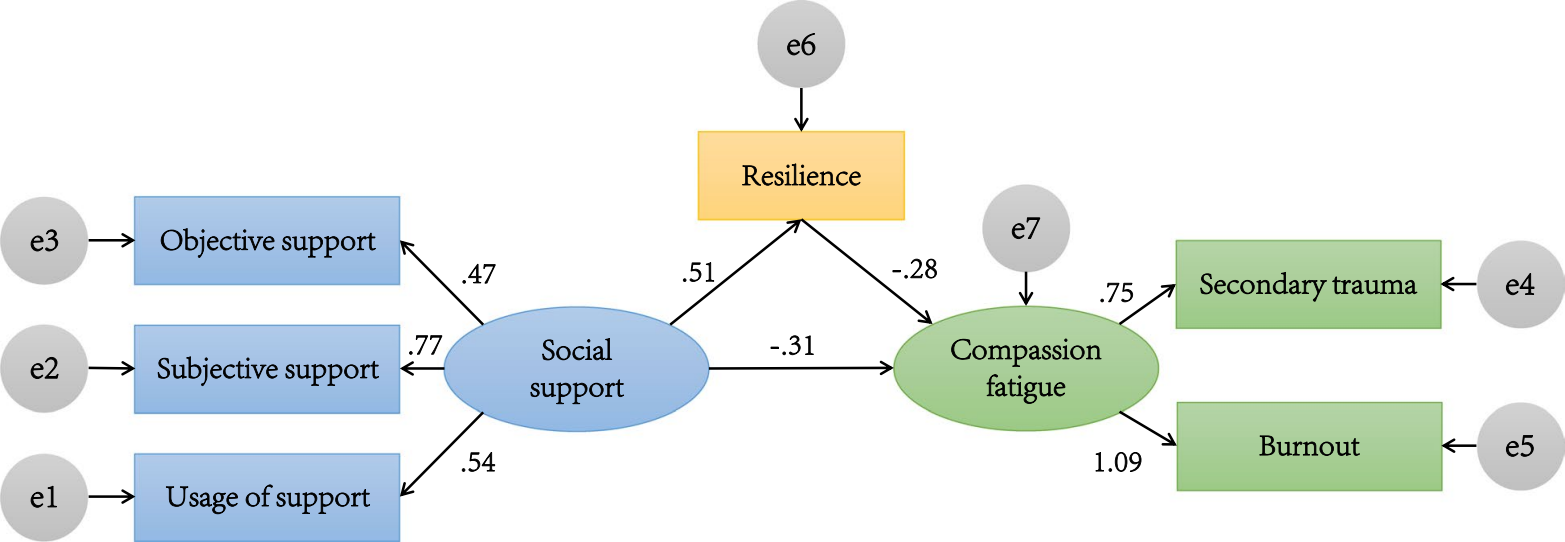
Structural equation modeling results. The structural model has adequate fit to the data. All the coefficients in this figure are standardized and significant at level 0.05

The following structural equation model was selected according to the principles of accuracy and conciseness. Fig. 1 shows the model of social support and resilience among intern nursing and midwifery students and its effect on compassion fatigue. Social support was considered an independent variable, compassion fatigue was taken as a dependent variable, and resilience was considered an intermediary variable for constructing a structural equation model and testing its hypothesized relation. The factor loadings between the latent variables and the respective observed variables in this model were 0.47–1.09; the path coefficients among social support and resilience, resilience and compassion fatigue, social support and compassion fatigue were 0.51, − 0.28, and − 0.31, respectively. All path coefficients in the model were statistically significant (*p* < 0.05), and the measurement model was not modified by deleting any variables. The fitting index results of the model showed that the initial model presented a satisfactory level of fit, with χ^2^/*df* = 1.744, RMSEA = 0.049, GFI = 0.987, AGFI = 0.962, NFI = 0.980, IFI = 0.992, TLI = 0.982 and CFI = 0.991.

Hypothesis 1 was supported. Social support had a positive direct effect on resilience (*β* = 0.514, *p* < 0.001, 95%CI = (confidence interval) [0.395, 0.620]). Hypothesis 2 was supported. Resilience had a negative direct effect on compassion fatigue (*β* = − 0.283, *p* < 0.001, 95% CI = [− 0.419, − 0.135]). Hypothesis 3 was supported in this model. It was found that social support had a negative direct and total effect on compassion fatigue (*β *= − 0.310, *p* < 0.001, 95% CI = [− 0.447, − 0.191]; *β* = − 0.455, *p* < 0.001, 95% CI = [− 0.570, − 0.343]). Furthermore, social support had an indirect effect on compassion fatigue (*β* = − 0.145, *p* < 0.001, 95% CI = [− 0.229, − 0.076]) via resilience, which supported Hypothesis 4. Table 4 represents the specific effect values of each path in this model. Therefore, the hypothesis model of this study fit the data well, and the results showed that social support positively affected resilience, while resilience negatively affected compassion fatigue. It was also found that resilience mediated between social support and compassion fatigue, and the significant mediation effects were partial.

**Table 4:** The total effects, direct effects, and indirect effects of each path in this model

		BC 95% CI ^**a**^		
Estimate	***β***	Lower	Upper	***p***
**Total effects**				
Resilience → Compassion fatigue	− 0.283	− 0.419	− 0.135	< 0.001
Social support → Compassion fatigue	− 0.455	− 0.570	− 0.343	< 0.001
**Direct effects**				
Social support → Resilience	0.514	0.395	0.620	< 0.001
Resilience → Compassion fatigue	− 0.283	− 0.419	− 0.135	< 0.001
Social support → Compassion fatigue	− 0.310	− 0.447	− 0.191	< 0.001
**Indirect effects**				
Social support → Resilience → Compassion fatigue	− 0.145	− 0.229	− 0.076	< 0.001

^a^ Means that 95% bias-corrected bootstrap confidence interval

## Discussion

To our knowledge, this is the first study to explore the mediating effects of resilience between social support and compassion fatigue among intern nursing and midwifery students during COVID-19. The prevalence of compassion fatigue is increasing compared to the prepandemic period [43, 44]. During the study period (from November 1st, 2020, to February 28th, 2021), moderate compassion fatigue was observed among intern nursing and midwifery students. This level of emotional stress was consistent with an earlier study conducted among Chinese nursing students [6] but higher than a study conducted in Chinese junior college intern nursing students from December 2021 to June 2022 [45]. Therefore, a decline in the degree of compassion fatigue was observed with the declining prevalence of COVID-19. In China, undergraduate nursing education emphasizes humanistic care, and students are encouraged to put themselves in the patient’s shoes, which successfully instilled deep empathy [46]. However, witnessing patients’ death was a tremendous shock to fourth-year college students, as they lacked clinical experience, solid knowledge, and skills [47, 48]. Their psychological immaturity interfered with adequate expression of empathy. Thus, they may be similarly vulnerable to the deleterious effects of compassion fatigue as professional nurses.

The results of this study showed that intern nursing and midwifery students had lower social support scores than midwives or nurses in prior studies [49, 50]. The rotation-based internship model is not conducive to the development of a support network [21]. In addition, relationships with teachers, family members, and peers were limited by social distancing measures. In this study, the majority of participants were females, who were more dependent on social support than males [51, 52]. As hypothesized, social support had a negative effect on compassion fatigue among intern nursing and midwifery students, consistent with a previous study [6]. Notably, objective support was not significantly correlated with compassion fatigue. This may be because objective support is typically visible or practical support, while compassion fatigue is emotionally driven. Hence, nurse educators should seek multiple sources of social support, especially peer support, for intern nursing and midwifery students.

Our observations on resilience were in line with previous studies [53–55]. Compared to the prepandemic period, COVID-19 resulted in increased stress among intern nursing and midwifery students, with stressors including uncertainty about unknown events, fear of medical errors, heavy academic and clinical workloads, and the gap between theory and practice [56]. This might explain the low resilience scores, which ultimately affected intern nursing and midwifery students’ mental health [57]. As hypothesized, resilience had a negative effect on compassion fatigue among intern nursing and midwifery students. Resilience is also critical to enable nurses to succeed in unpredictable nursing practice [58, 59]. This finding urged intern nursing and midwifery students to learn to be flexible in responding to different stressors, multitask effectively and balance academic and professional workloads.

The most striking finding of our study was an important mediating role of resilience between social support and compassion fatigue among intern nursing and midwifery students. It emphasized the mediating effects of resilience as positive psychological resources in line with previous studies [60, 61]. This study enriched the knowledge on mediating mechanisms of resilience, explaining associations between social support and compassion fatigue among intern nursing and midwifery students. Resilience was suggested as an important weapon in the arsenal against compassion fatigue [62]. Resilience helps assuage unpleasant feelings and prevent individuals from developing psychological problems [63]. Additionally, mindfulness training, stress management skills, and communication skills were among the strategies examined to promote resilience [64]. It is high time that educators focus on the mental health of intern nursing and midwifery students, and by incorporating resiliency training, students can be prepared for nursing practice.

### Limitations

Several limitations should be considered in interpreting the results. First, the study used convenience sampling, and all the participants in our study were selected only from tertiary hospitals in the eastern coastal city province. It is therefore not representative of the whole intern nursing and midwifery student population in China. Future research should test the correlation in more representative samples from multiple regions and manage to collect data from multiple sources. Second, numerous factors influenced compassion fatigue, and the current study only examined resilience as a mediating variable. Social support and resilience could explain only a limited part of compassion fatigue. Hence, future studies should focus on a more integrated model of compassion fatigue with diverse influencing factors. Interventions based on resilience to reduce compassion fatigue should be developed.

## Conclusion

In conclusion, intern nursing and midwifery students had a moderate level of compassion fatigue. Resilience plays a mediating role in the development process from social support to compassion fatigue among intern nursing and midwifery students. Resilience can be a useful resource for improving psychological well-being. Therefore, nurse educators should provide strategies to help students maintain or grow resiliency during the COVID-19 pandemic.

## Supplementary Information

**Additional file 1:** **Appendix S1.** STROBE Statement—EQUATOR checklist of items that should be included in reports of observational studies.

## Data Availability

The dataset supporting the conclusions is available from the corresponding author on reasonable request.
